# Clusters of facilitatory and inhibitory conditioned pain modulation responses in a large sample of children, adolescents, and young adults with chronic pain

**DOI:** 10.1097/PR9.0000000000001032

**Published:** 2022-10-04

**Authors:** Don Daniel Ocay, Diana-Luk Ye, Cynthia L. Larche, Stéphane Potvin, Serge Marchand, Catherine E. Ferland

**Affiliations:** a Department of Experimental Surgery, Faculty of Medicine and Health Sciences, McGill University, Montreal, QC, Canada; b Clinical Research Department, Shriners Hospitals for Children, Montreal, QC, Canada; c Child Health and Human Development research axis, Research Institute of the McGill University Health Centre, Montreal, QC, Canada; d Department of Psychiatry, Faculty of Medicine, Université de Montréal, Montreal, QC, Canada; e Centre de recherche du Centre Hospitalier de l’Université de Sherbrooke, Sherbrooke, QC, Canada; f Department of Anesthesia, Faculty of Medicine and Health Sciences, McGill University, Montreal, QC, Canada

**Keywords:** Cluster, Pediatric, Conditioned pain modulation, Chronic pain, Control

## Abstract

**Introduction:**

When investigating the role of facilitatory and inhibitory pain mechanisms such as conditioned pain modulation (CPM) and temporal summation of pain (TSP), it is important to take both into consideration in a single experimental model to provide the most information on subgroups of patients. Therefore, the objective of this study was to identify subgroups in a large population of pediatric patients with chronic pain based on their facilitatory and inhibitory pain mechanisms and compare them with control subjects.

**Methods:**

Five hundred twenty-one female subjects and 147 male subjects between 8 and 21 years old underwent a CPM assessment using a 2-minute tonic noxious heat stimulation as the test stimulus and a 2-minute cold-pressor task (CPT) (12°C) as the conditioning stimulus.

**Results:**

The best partition of clusters of patients was 3 clusters accounting for 27.15% of the total variation in the data. Cluster 1 (n = 271) was best characterized by high pain intensity during the CPT, lack of TSP during the test stimuli, and efficient inhibitory CPM. Cluster 2 (n = 186) was best characterized by low pain intensity during the CPT, lack of TSP during the test stimuli, and efficient inhibitory CPM. Cluster 3 (n = 151) was best characterized by high pain intensity during the CPT, presence of TSP during the test stimuli, and inefficient inhibitory CPM.

**Discussion:**

A single thermal CPM experimental design can identify combinations of facilitatory and inhibitory pain modulation responses. Findings from the current study add to the literature by describing different clinical phenotypes of central pain mechanisms of youth with chronic pain.

## Abstract 2

Supplemental Digital Content is Available in the Text.

Findings from the current study add to the literature by describing different clinical phenotypes of central pain mechanisms of youth with chronic pain.

## 1. Introduction

Chronic pain affects about 11% to 38% of youth.^18^ Using psychophysical procedures, pediatric studies have shown that chronic pain is associated with altered excitatory and inhibitory endogenous pain modulation systems.^2,7,8,16,17,22,23,38,40,50^ The endogenous inhibitory pathways of pain modulation can be indirectly assessed using a conditioned pain modulation (CPM) paradigm using the concept of “pain inhibits pain,” in which one painful stimulus, the conditioning stimulus (CS), modulates another pain-inducing stimulus, the test stimulus (TS).^17,26^ Studies have observed a lower capacity to inhibit the post-conditioned painful TS in patients with chronic pain conditions when compared with age-matched control subjects.^16,22,29,40,52^ The endogenous facilitatory pain modulation mainly assessed using a temporal summation paradigm have been shown to be involved in some chronic pain conditions.^2,6,8,38,39,50^ Temporal summation of pain (TSP) is referred to as an amplification of pain perception in response to repeated or continuous painful stimulation, at a constant intensity, which indirectly reflect an increased excitability at the spinal level and receptive fields of the nociceptive spinal cord neurons.^34^ Evaluating temporal summation will help understand the endogenous facilitatory pain mechanisms (eg, central sensitization) in youth and its role in chronic pain conditions.

Considering the role of endogenous facilitatory and inhibitory pain responses such as CPM and TSP, and the heterogeneity within the different populations, it is important to take both into consideration in a single experimental model to give as much information as possible on subgroups of patients that may benefit from a specific therapeutic treatment.^53^ Researchers and clinicians have turned to identify distinct subgroups of pediatric chronic pain patients that may be relevant for treatment because individuals respond differently to standardized treatments.^35,37,49,50^ However, these studies strictly investigated pain and psychosocial characteristics in their analysis and there is limited data evaluating subgroups based on the endogenous pain mechanisms of pediatric chronic pain patients. Our group has shown the heterogeneity of CPM efficiency and temporal summation in samples of patients with chronic musculoskeletal pain.^12,27,28,40^ However, the pain modulation responses were considered separately and no association between facilitatory and inhibitory pain modulation responses were investigated or observed.

Therefore, the objective of this study was to identify subgroups in a large population of pediatric patients with chronic pain based on their endogenous facilitatory and inhibitory pain modulation responses. We conducted an exploratory analysis investigating interrelationships between individuals regarding their CPM efficiency and TSP from one CPM experimental design.

## 2. Methods

### 2.1. Participants

This study regrouped multiple studies whose ethics approval were all obtained before the beginning of the recruitment from the McGill University and McGill University Health Centre Research Ethics Boards (A08-M71-14B, A11-M62-15B, A09-M17-17B, 2019-4887, see Supplementary Table 1 for more details on sample, available at http://links.lww.com/PR9/A172). This has facilitated analysis of a large and novel cohort for investigation unlike our previously published work.^12,27,28,40^ Between 2015 and 2021, patients were recruited in the spine or orthopedic outpatient clinics of the Shriners Hospitals for Children—Canada or by referral from the Chronic Pain Clinic from the Montreal Children's Hospital. Age-matched control subjects with no chronic pain were recruited between 2018 and 2021 through word of mouth, advertisements, and a collaborative high school nearby. Signed informed consent was obtained from participants over 14 years old and parents of participants aged 13 years and younger. To ensure appropriate comparison across the different studies, appropriate inclusion or exclusion criteria for the patients included in the analysis were established. Inclusion criteria for patients were male or female between 8 and 21 years old, reporting chronic primary or secondary pain (at least once a week for more than 3 months). Participants who did not speak English or French or had developmental delay or substantial functional limitations that would interfere with completing measures were excluded from the study.

### 2.2. Conditioned pain modulation assessment

#### 2.2.1. Pain perception

Pain before the assessment was measured verbally using a numerical rating scale (NRS) ranging from 0 (no pain) to 10 (worst pain imaginable). Pain perception during the heat pain procedure was assessed using a computerized visual analogue scale (CoVAS), ranging from 0 (no pain) to 100 (worst pain imaginable), linked to a 9-cm^2^ warm calibrated thermode connected to a Q-sense apparatus (Medoc, Israel). Pain perception during the cold pain procedure was assessed verbally using the NRS of 0 to 10.

#### 2.2.2. Pretest

Conditioned pain modulation assessment was conducted using a protocol as previously described by our group.^12,27,28,40^ Tests were conducted by research assistants, who were trained and evaluated by the principal investigator of the study, following rigorous standards of procedure to decrease between-tester variability. The thermode with a baseline of 32°C and a 0.3°C/s upslope was applied 3 times. Participants were given the CoVAS and advised to move the cursor towards the “100” mark when they first report pain (pain threshold) and that the cursor had to be at the “100” mark when the pain was intolerable. The mean temperature at which they rated their pain intensity at 50/100 with the CoVAS was calculated.

#### 2.2.3. Test stimulus

The thermode was applied to the right forearm to reach a predetermined test temperature to a pain intensity 50/100 (T50) and it remained constant for 120 seconds. Participants were told to evaluate their pain with the COVAS throughout the test. The average pain intensity during the 120 seconds was calculated.

#### 2.2.4. Conditioning stimulus

A cold-pressor task (CPT) was used as the CS involving the immersion of their left forearm in a bath filled with cold water (12°C) for 120 seconds. Every 15 seconds, the participants verbally reported their pain intensity using the NRS of 0 to 10. The average pain intensity during the CS was then calculated. If pain was intolerable, participants could remove their arm before the end of the 120 seconds, and an average pain intensity score of 10/10 was given.

### 2.3. Assessment of inhibitory pain response

To evaluate the endogenous inhibitory pain response (CPM efficiency), the CPT was immediately followed by a second tonic heat TS with the same predetermined test temperature. Pain modulation was measured as the percentage difference in average pain intensity of the test stimuli^54^: 100% × (CoVAS_after_ − CoVAS_before_)/CoVAS_before_. A CPM efficiency between −100% and −30% was considered optimal, between −30% and −10% as suboptimal, and between −10% and +100% as inefficient. A 30% reduction in pain intensity was labelled to be a clinically important difference^11^ and is approximately the mean value of inhibitory CPM observed in previous studies.^12,32,40,42^

### 2.4. Assessment of facilitatory pain response

Facilitatory pain response (TSP) was assessed as the absolute difference in pain intensity during the last 60 seconds of each TS (temporal summation phase).^42^ An increase or decrease in pain intensity was determined clinically significant if the change was equal to or larger than 20/100 during the temporal summation phase.^11,45^

### 2.5. Statistical analysis

Statistical analyses were performed using the R Studio software. Data were assessed for normality and descriptive statistics were conducted to describe the sample and presented as mean ± SD, unless indicated otherwise. One-way analysis of variance (ANOVA) was conducted to determine differences in CPM assessment outcomes between gender, duration of chronic pain, presence of more than one pain site, and presence of pain before CPM assessment. Spearman correlation was conducted to determine whether age, pain before the assessment, and T50 were associated with the CPM assessment outcomes. Differences between patients and control subjects were determined using the χ^2^ test and one-way ANOVA controlling for gender because of gender differences observed in heat pain threshold (Supplementary Table 1, available at http://links.lww.com/PR9/A172), followed by the Scheffé test. The effect size (ω^2^) for significant ANOVA models was also calculated (small = 0.01; medium = 0.06; and large = 0.14). Clusters within the chronic pain sample were identified using an unsupervised clustering method performed using the FactoMineR package.^19^ To investigate the facilitatory and inhibitory pain modulation responses, the cluster analysis involved 4 quantitative indicator variables: (1) the absolute change in pain intensity during the last 60 seconds of the first TS (TS1); (2) the average pain intensity during the CS; (3) the absolute change in pain intensity during the last 60 seconds of the second TS (TS2); and (4) the CPM efficiency. Because of the different scales and units for each variable, hierarchical clustering with k-means consolidation was conducted on the 4 variables standardized into z-scores to ensure that all variables were considered equally. The best partition of clusters was the one with the highest relative loss of inertia^15^ and based on parsimony.

To determine cluster effect of the indicator variables, an ANOVA model was conducted along with a Fisher test. Differences between clusters and control subjects were conducted using the χ^2^ test and one-way ANOVA controlling for gender followed by Scheffé test.

## 3. Results

Six hundred thirty-nine patients and 60 control subjects consented. However, only 608 patients were included in the analysis (n = 31 did not complete the CPM assessment or had missing information from the CPM assessment). The mean age for patients was 15.18 ± 2.14 years (range = 8.2–21.4 years) and 80.92% were females. The mean age for control subjects was 15.06 ± 2.23 years (range = 10.0–18.9 years) and 48.33% were females. Most of the patients experienced persistent pain (n = 329) than recurrent pain (n = 223) for more than 6 months (n = 568) and primarily in their back (n = 410). The primary location of pain of the other patients included the head or neck (n = 31), the abdomen (n = 24), the groin area (n = 1), the thorax (n = 14), the upper extremities (n = 18), and the lower extremities (n = 109). Moreover, 50.99% of the patients reported more than one pain site. Before the assessment, 70.23% of the patients reported pain with a mean pain intensity of 4.16 ± 2.16. Overall, patients reported a mean pain intensity of 2.95 ± 2.62 (range = 0–10), with patients recruited from the pain clinic reporting significantly higher pain intensity before the CPM assessment (3.51 ± 2.61) than patients from the outpatient clinics (2.54 ± 2.56, *t* = 4.57, *P* < 0.001). Only one control subject reported mild pain before the CPM assessment.

**Figure 1.**
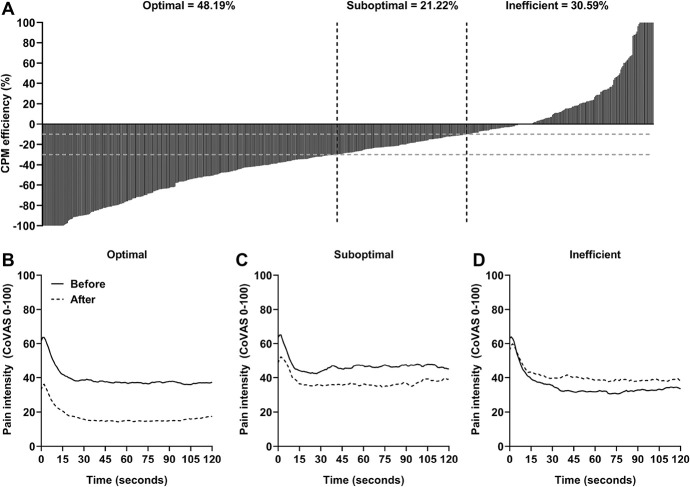
(A) Distribution of conditioned pain modulation (CPM) efficiency of the patient sample in which each bar represents one patient (n = 608). A negative value represents pain inhibition, whereas a positive value represents pain facilitation. The gray dotted lines mark the cutoffs for optimal (n = 293), suboptimal (n = 129), and inefficient (n = 186) CPM efficiency. (B–D) Mean pain intensity during the tonic thermal heat stimulations of the patients based on the different patterns of the CPM score: (B) optimal, (C) suboptimal, and (D) inefficient. A greater percentage difference in pain intensity during the tonic thermal heat stimulations demonstrates a greater CPM efficiency. CoVAS, computerized visual analog scale.

The average heat pain threshold was 38.95 ± 3.13°C and 38.71 ± 2.63°C for patients and control subjects, respectively (F = 1.17, *P* = 0.280). The average test temperature was 43.41 ± 2.38 and 42.84 ± 2.38°C for patients and control subjects, respectively (F = 4.33, *P* = 0.038, ω^2^ < 0.01). Heterogeneity within our patient sample was observed regarding the CPM efficiency (Fig. 1). The mean CPM efficiency for patients was −26.13% ± 43.20%. The mean CPM efficiency for control subjects was −32.47% ± 35.47% and was not significantly different from patients (F = 2.21, *P* = 0.137).

**Figure 2.**
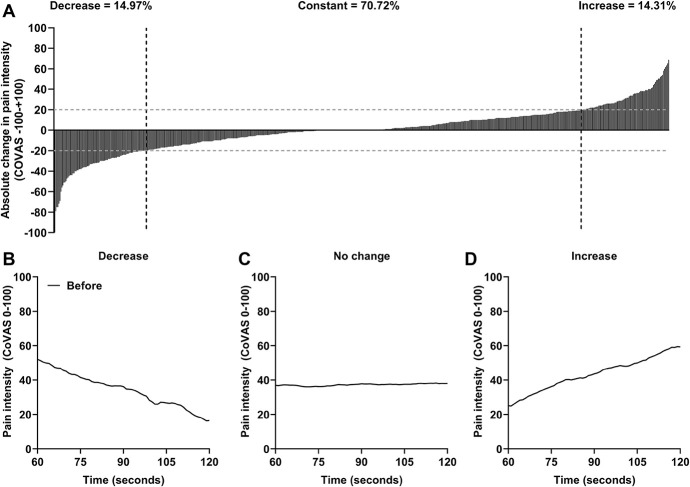
(A) Distribution of the change in pain intensity during the tonic thermal heat stimulation before the conditioning stimulus (TS1) of the patient sample in which each bar represents one patient (n = 608). The gray dotted line marks the cutoffs for a significant decrease of −20/100 (n = 91), no change (n = 430), and a significant increase of 20/100 (n = 87) in pain intensity. (B–D) Mean pain intensity during the last 60 seconds of the tonic thermal heat stimulation before the conditioning stimulus of the patients based on the different patterns of change in pain intensity: (B) a decrease, (C) no change, or (D) an increase in pain intensity. CoVAS, computerized visual analog scale.

**Figure 3.**
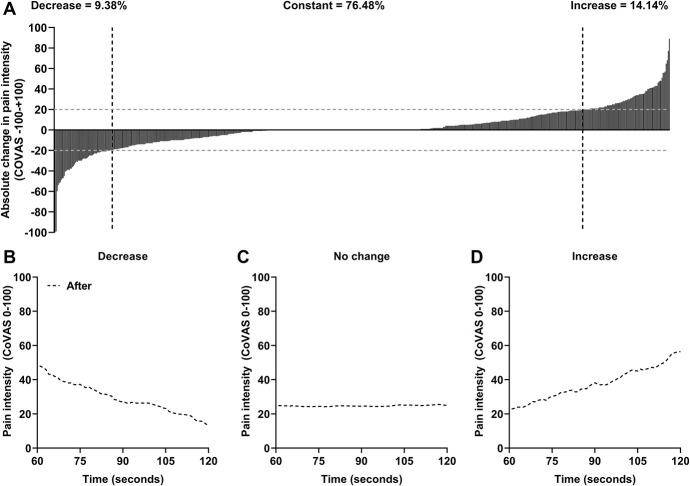
(A) Distribution of the change in pain intensity during the tonic thermal heat stimulation after the conditioning stimulus (TS2) of the patient sample, in which each bar represents one patient (n = 608). The gray dotted line marks the cutoffs for a significant decrease of −20/100 (n = 57), no change (n = 465), and a significant increase of 20/100 (n = 86) in pain intensity. (B–D) Mean pain intensity during the last 60 seconds of the tonic thermal heat stimulation after the conditioning stimulus of the patients based on the different patterns of change in pain intensity: (B) a decrease, (C) no change, or (D) an increase in pain intensity. CoVAS, computerized visual analog scale.

Heterogeneity within patients was also observed regarding the change in pain intensity during the last 60 seconds of TS1 (Fig. 2) and TS2 (Fig. 3). The mean reported change in pain intensity during the last 60 seconds of TS1 was 0.45 ± 21.70 in our patients and was significantly different from control subjects, whose mean reported change in pain intensity was 6.46 ± 19.05 (F = 4.92, *P* = 0.027, ω^2^ = 0.01). The mean reported change in pain intensity during the last 60 seconds of TS2 was 1.84 ± 19.05 in our patients, but was not significantly different from control subjects, whose mean reported change in pain intensity was 5.16 ± 14.49 (F = 1.63, *P* = 0.202).

The mean reported pain intensity during the CS was 6.92 ± 2.44 and 6.31 ± 2.41 for patients and control subjects, respectively (F = 4.03, *P* = 0.027, ω^2^ < 0.01). Fifty-one patients and 6 control subjects removed their arm before the end of the 120 seconds. However, no difference in CPM efficiency was observed between the participants who completed the CPT and those who did not (data not shown).

A significant positive association was observed between the age of participants and their T50 (rho = 0.137, 95% CI = 0.059–0.212, *P* < 0.001) and their mean pain intensity during the CPT (rho = −0.086, 95% CI = −0.163–0.008, *P* = 0.027). Furthermore, a significant positive association was observed between the participants' T50 and the change in pain intensity during the temporal summation phase of the TS before (rho = 0.205, 95% CI = 0.129–0.279, *P* < 0.001) and after (rho = 0.218, 95% CI = 0.142–0.291, *P* < 0.001) the CPT. Other within-cohort differences or associations can be found in Supplementary Table 2 (available at http://links.lww.com/PR9/A172).

### 3.1. Cluster analysis

**Figure 4.**
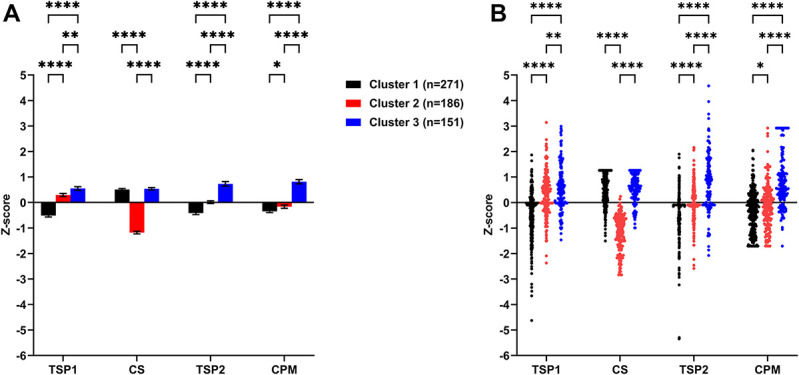
(A) Bar and (B) scatter plot of the indicator variables respective of the 3 clusters derived from the hierarchical cluster analysis with k-means. A score of zero is aligned with the mean of the sample. The *P* values of Fisher pairwise comparisons are shown. *P < 0.05; **P < 0.01; ***P < 0.05; ****P < 0.01. Bars = mean ± SEM. Points = individual patients. TSP1, change in pain intensity during last 60 seconds of the first test stimulus; CS, conditioning stimulus; TSP2, change in pain intensity during last 60 seconds of the second test stimulus; CPM; conditioned pain modulation efficiency. Patients grouped in cluster 1 are characterized by significantly lower values for TSP1, TSP2, and CPM efficiency compared with cluster 2 and 3. Patients grouped in cluster 3 are characterized by significantly higher values for TSP1, TSP2, and CPM efficiency compared with cluster 1 and 2. Patients in cluster 2 are characterized to have significantly lower values for CS compared with cluster 1 and 3.

The best partition of clusters of the patient sample was 3 clusters accounting for 27.15% of the total variation in the data (Fig. 4). 271 patients (44.57%) were grouped in cluster 1, 186 (30.59%) in cluster 2, and 151 (24.84%) in cluster 3.

No significant between-cluster difference was observed regarding their demographic characteristics (Table 1). However, significant differences were observed between clusters and control subjects regarding all CPM-related outcomes (ω^2^ ranging from 0.05 to 0.56) (Table 2).

**Table 1:** Demographic characteristics of each cluster.

Variable	Cluster 1 (n = 271)	Cluster 2 (n = 186)	Cluster 3 (n = 151)	Test statistic	*P*
Location of recruitment, n (%)				3.01*	0.222
Chronic pain clinic	106 (39.11)	81 (43.55)	72 (47.68)		
Orthopedic outpatient clinic	165 (60.89)	105 (56.45)	79 (52.32)		
Age, mean ± SD (range)	15.15 ± 2.12 (8.2–21.4)	15.26 ± 1.86 (9.0–19.3)	15.15 ± 2.49 (8.3–21.0)	0.21†	0.810
Gender, n (%)				1.84*	0.398
Female	218 (80.44)	156 (83.87)	118 (78.15)		
Male	53 (19.56)	30 (16.13)	33 (21.85)		
Duration of chronic pain, n (%)				1.93*	0.381
3–6 mo	18 (6.64)	9 (4.84)	13 (8.61)		
More than 6 mo	253 (93.36)	177 (95.16)	138 (91.39)		
Type of chronic pain, n (%)				4.84*	0.089
Persistent	132 (48.71)	109 (58.60)	88 (58.28)		
Recurrent	110 (40.59)	66 (35.48)	47 (31.13)		
Primary location of pain, n (%)				19.00*	0.165
Head/neck	14 (5.17)	13 (6.99)	4 (2.65)		
Upper limbs	4 (1.48)	10 (5.38)	4 (2.65)		
Thorax	6 (2.21)	6 (3.23)	2 (1.32)		
Abdomen	12 (4.43)	9 (4.84)	3 (1.99)		
Back	192 (70.85)	113 (60.75)	105 (69.53)		
Groin	1 (0.37)	0	0		
Lower limbs	41 (15.13)	35 (18.82)	33 (21.85)		
Presence of secondary pain sites, n (%)				2.89*	0.236
No	139 (51.29)	94 (50.54)	65 (43.04)		
Yes	132 (48.71)	92 (49.46)	86 (56.96)		
Presence of pain before CPM assessment, n (%)				1.32*	0.516
No	79 (27.15)	49 (26.34)	48 (31.79)		
Yes	188 (69.38)	137 (73.66)	102 (67.55)		
Average pain intensity, mean ± SD (range)	3.08 ± 2.78 (0–10)	2.94 ± 2.50 (0–10)	2.73 ± 2.48 (0–8.5)	0.81†	0.444

* χ^2^ test statistic.

† One-way ANOVA test statistic controlled for gender.

**Table 2:** Facilitatory and inhibitory pain responses of each cluster and control subjects.

Variable	Cluster 1 (n = 271)	Cluster 2 (n = 186)	Cluster 3 (n = 151)	Control Subjects (n = 60)	Test statistic	*P*	ω^2^ value
Heat pain threshold (°C), mean ± SD	38.23 ± 2.90^b^	39.98 ± 3.33^a,c^	38.96 ± 2.92^b^	38.71 ± 2.63^b^	13.39*	<0.001	0.05
Test temperature (°C), mean ± SD	42.70 ± 2.45^b,c^	44.39 ± 1.98^a,c^	43.49 ± 2.27^a,b^	42.84 ± 2.38^b^	22.04*	<0.001	0.09
Change in pain intensity during the last 60 s of TS1 (NRS −100 to +100), mean ± SD	−10.62 ± 20.17^b,c^	6.89 ± 17.72^a^	12.39 ± 19.23^a^	6.46 ± 19.05^a^	58.54*	<0.001	0.21
Decrease, n (%)	73 (26.94)	12 (6.45)	6 (3.31)	5 (8.33)	97.73†	<0.001	
Constant, n (%)	189 (69.74)	139 (74.73)	102 (67.55)	43 (71.67)			
Increase, n (%)	9 (3.32)	35 (18.82)	43 (28.48)	12 (20.00)			
Average pain intensity during CS (NRS 0–10), mean ± SD	8.16 ± 1.47^b^	4.04 ± 1.70^a,c^	8.24 ± 1.32^b^	6.31 ± 2.41^a,b,c^	287.23*	<0.001	0.56
Change in pain intensity during the last 60 s of TS2 (NRS −100 to +100), mean ± SD	−6.11 ± 16.42^b,c^	2.10 ± 14.11^a,c^	15.78 ± 20.66^a,b^	5.16 ± 14.49^a,c^	55.90*	<0.001	0.2
Decrease, n (%)	39 (14.39)	11 (5.91)	7 (4.64)	4 (6.67)	110.15†	<0.001	
Constant, n (%)	222 (81.92)	157 (84.41)	86 (56.95)	47 (78.33)			
Increase, n (%)	10 (3.69)	18 (9.68)	58 (38.41)	9 (15.00)			
CPM efficiency (%), mean ± SD	−41.06 ± 34.19^c^	−33.10 ± 37.84^c^	9.25 ± 44.27^a,b^	−32.67 ± 35.47^c^	60.87*	<0.001	0.21
Inefficient, n (%)	45 (16.61)	44 (23.66)	97 (64.24)	14 (23.33)	116.79†	<0.001	
Suboptimal, n (%)	61 (22.51)	44 (23.66)	24 (15.89)	12 (20.00)			
Optimal, n (%)	165 (60.89)	98 (52.69)	30 (19.87)	34 (56.67)			

a–c: Significant difference through Scheffé post hoc test (*P* < 0.05) from cluster 1 to cluster 3, respectively; ω^2^ value: 0.01 (small), 0.06 (medium), and 0.14 (large); TS1, tonic thermal heat stimulation before the conditioning stimulus; CS, conditioning stimulus; TS2, tonic thermal heat stimulation after the conditioning stimulus; CPM, conditioned pain modulation.

* One-way ANOVA test statistic controlled for gender.

† χ^2^ test statistic.

**Figure 5.**
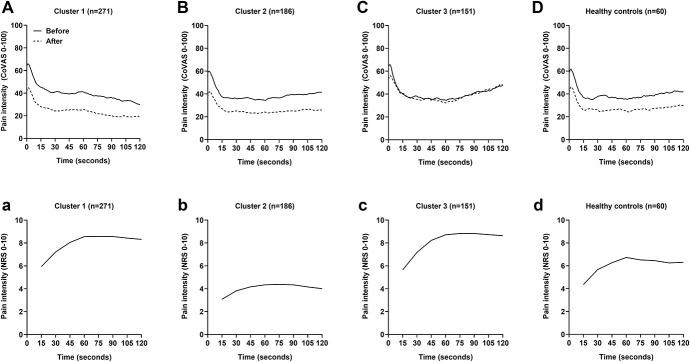
Mean pain intensity during the tonic thermal heat stimulations (A–D) and cold-pressor task (a–d) for each patient cluster and healthy control subjects. CoVAS, computerized visual analog scale; NRS, numerical rating scale.

Patients in cluster 1 significantly displayed the lowest test temperature used for the TS and a higher proportion displayed a significant decrease in pain intensity (ie, −20/100) during the temporal summation phase of TS1 and TS2 (Fig. 5A). Patients in cluster 2 significantly displayed the highest test temperature used for the TS and the lowest average pain intensity reported during the CS. Interestingly, despite a large proportion of this cluster displaying optimal CPM efficiency similar to cluster 1, a larger proportion displayed a significant increase in pain intensity (ie, +20/100) during the temporal summation phase of TS1 and TS2 than cluster 1 (Fig. 5B). In contrast to cluster 1, patients grouped in cluster 3 significantly displayed a higher test temperature used for the TS, but lower than cluster 2, and a higher proportion displayed a significant increase in pain intensity during the temporal summation phase of TS1 and TS2 (Fig. 5C). Moreover, a larger proportion of cluster 3 displayed an inefficient CPM.

When the clusters were compared with the control subjects, a significant difference in heat pain threshold (*P* = 0.025) and T50 (*P* < 0.001) was observed between cluster 2 and the control subjects. The heat pain threshold and T50 of control subjects were significantly lower than patients in cluster 2. A higher proportion of control subjects displayed a significant increase in pain intensity during the last 60 seconds of TS1 and TS2 than patients in cluster 1 (*P* < 0.001). However, a smaller proportion of control subjects displayed a significant increase in pain intensity during the last 60 seconds of TS2 than patients in cluster 3 (*P* = 0.002). The average pain intensity of control subjects during the CPT was significantly different to all clusters. When controlling for gender, the average pain during the CPT of patients in cluster 2 was significantly lower than control subjects (*P* < 0.001), whereas the average pain during the CPT of patients in cluster 1 (*P* < 0.001) and 3 (*P* < 0.001) was significantly higher than control subjects (Fig. 5D). The mean CPM efficiency of control subjects was optimal and significantly different (*P* < 0.001) from patients in cluster 3, which was inefficient.

## 4. Discussion

The objective of this study was to identify subgroups of patients with chronic pain based on their endogenous pain mechanisms. This analysis revealed heterogeneity in our patients regarding their facilitatory and inhibitory pain responses from one experimental design. We observed a significant association between the T50 and the age of the participants, and the change in pain intensity during the temporal summation phase of the TS. Furthermore, based on the CPM assessment outcomes of the patients, 3 subgroups were identified to best describe the patients. Cluster 1 was best characterized by high pain intensity during the CPT, lack of TSP, and efficient inhibitory CPM. Cluster 2 was best characterized by low pain intensity during the CPT, lack of TSP, and efficient inhibitory CPM. Cluster 3 was best characterized by high pain intensity during the CPT, presence of TSP, and inefficient inhibitory CPM.

A weak positive correlation was observed between the test temperature of the test stimuli of the participants and their age. Research in small samples of healthy children and adolescents or patients with type 1 diabetes mellitus have observed no correlation between age and heat-induced pain threshold.^1,47^ However, Blankenburg et al.^5^ observed a strong effect of age on heat pain threshold in a large population of healthy children and adolescents. Our findings extend their observation by demonstrating in a large population of pediatric sample with or without chronic pain that younger children are more sensitive to heat-induced pain. Age and sex have been shown to impact CPM in adult populations, such that male subjects have a greater CPM efficiency than female subjects, and older adults show less CPM.^31^ Only the effect of age has been observed in healthy youth, such that older children (12–17 years) showed greater CPM efficiency compared with younger children (8–11 years).^44^ In the current study, no association was observed between age or sex and CPM efficiency, and no age/sex differences were observed between clusters. Although it has been hypothesized that pain inhibitory mechanisms may develop throughout childhood and become stronger during adolescence, other predetermining factors may moderate the effect of age on CPM efficiency.

A significant difference in the average pain intensity reported during the CPT was observed between the patients and control subjects. The literature has shown conflicting results regarding pain responsivity in children with chronic pain during the CPT compared with control subjects.^9,20,41,43^ Because of individual variability in pain perception, a group difference in pain perception in previous studies with smaller sample sizes may be difficult to detect. Our large sample found a significant effect between groups that is small in magnitude but was more evident after cluster analysis, where patients grouped in cluster 1 and 3 reported significantly higher pain intensity during the CPT. Several aspects of the CPT methodology may also explain the conflicting results in the literature, such as CPT preparation, water temperature, immersion time, audience effects, arm removal, and measurement of pain outcomes.^4^ An advantage of the CPT is the opportunity to observe or explore the influence of psychosocial and cognitive factors on pain and to test new psychological interventions for pain.^30,48^ Holley et al. observed that higher state pain catastrophizing in youth with new-onset pain significantly predicted higher cold-pressor pain, but trait pain catastrophizing had an inverse relationship.^20^ This suggests that state and trait characteristics in our population of pediatric patients may have different patterns of relevance in their chronic and acute pain experiences and may explain why patients in cluster 1 and 3 displayed higher pain intensity during the cold-pressor task.

Patients grouped in cluster 3 displayed significant manifestation of impairment in central pain modulation, as observed in the presence of increased TSP during the test stimuli, and the large proportion of patients that displayed inefficient descending inhibitory pain control in this cluster, especially in comparison with control subjects. Studies in children and adolescents with chronic pain have observed overall lower inhibitory CPM response and facilitated temporal summation in comparison with control subjects.^17^ Walker et al.^50^ observed that a subgroup of pediatric patients with functional abdominal pain and met the criteria for functional gastrointestinal disorders at their follow-up appointment presented significantly greater thermal pain wind-up at their follow-up appointment, suggesting the involvement of central pain modulation in this transition. In our large population of patients, we observed a small proportion of patients (24.84%) displayed amplification in facilitatory pain mechanisms with impairment in inhibitory conditioned pain modulation responses. With such manifestation of impairment in central pain modulation, these patients are suggested to be at high propensity for widespread pain and comorbidities in the future.^46,53^ However, this was not investigated because of the cross-sectional nature of the analysis such that the long-term stability over weeks or months was not studied in this population. Neuronal plasticity occurs in children throughout development, which can shape the functional integrity of the descending inhibitory systems. A previous study in young children observed that prematurity and exposure to numerous painful interventions after birth lead to alterations in the endogenous pain modulatory mechanisms.^14^ Therefore, it is unknown whether patients shift from one cluster to another depending on multiple factors such as developmental neuroplasticity or if a therapeutic intervention was given.^21,51^

Unexpectedly, a significant difference was observed between patients and control subjects regarding the test temperature of the test stimuli, in which patients required higher temperature to induce pain intensity of 50/100. However, the effect size was very small, but this effect emerged nevertheless as being significantly different probably because of the large sample size that was recruited in the current study. The effect size became medium after cluster analysis was conducted, such that a significant difference in heat pain threshold and T50 was observed only between cluster 2 and control subjects. Studies using thermal modalities during CPM assessment in pediatrics display conflicting results between patients and control subjects regarding their heat pain threshold or test temperature.^20,22,52,55^ Thermal experimental heat pain through a thermode allows for predictable stimulations of pain with a sharp and piercing sensation with various durations.^3^ As thermal pain threshold and the T50 reflect the perception of acute pain, the fact we did not observe hyperalgesic responses in the pediatric chronic pain patients using these measures may not be fully surprising because they probably do not portray all mechanisms relevant to chronic pain. There is indeed evidence in the adult literature indicating that tonic noxious stimuli correlate better with clinical pain than acute stimuli because clinically relevant pain rarely lasts only for a few seconds.^13,25,36^ Pain normally lasts for minutes to hours or longer. It has been proposed that tonic stimulation paradigms seem better to investigate pain in more real-world circumstances by the fact that tonic noxious stimuli recruit endogenous pain modulation mechanisms.^13,25,36^

A significant difference was observed in the change in pain intensity during the temporal summation phase of the TS before the CS between our patients and control subjects. Unexpectedly, the change in pain intensity during the temporal summation phase of TS1 of control subjects was significantly higher than that of patients. However, the effect size was small. This statistical significance between cohorts was probably because of the large patient sample size. Moderate effects were only observed after cluster analysis was conducted. A study conducted by Potvin et al. observed lower temporal summation of pain in a large proportion of adult patients with fibromyalgia when compared with control subjects.^33^ However, the test temperature was significantly lower in patients with fibromyalgia, suggesting that hypersensitivity may have been present before the CPM assessment, which was not the case in our sample. Studies in children have shown conflicting results regarding the presence or absence of TSP in patients with chronic pain.^55,56^ However, these studies had a small sample size, meaning that the observed lack of significant differences may be because of a lack of statistical power (eg, type II error). Therefore, our results highlight that in a large sample of pediatric patients with chronic pain, there is only a subgroup of patients who display hyperexcitability of the central nervous system through TSP.

The generalizability of our findings to children, adolescents, and young adults with chronic pain should be interpreted considering certain limitations. Chronic pain is a dynamic and complex phenomenon influenced by many variables such as individual predisposition, pathology, psychological factors, and environmental factors.^9,10,14,24^ Most of the patients reported pain in their back because of the patients primarily being recruited from the spine outpatient clinics of our institution, and 2 of the 4 studies including only patients with spinal pathologies. Therefore, despite no between-cluster difference based on location of pain or other demographic and clinical variables, replication studies using a similar simple clustering method investigating facilitatory pain responses and inhibitory conditioned pain modulation responses alongside the medical history of patients, their psychosocial variables, and their physical functioning are warranted. Another limitation is the small sample of control subjects in our analysis. It is unknown if similar differences would have been observed if the control group would have been larger. Furthermore, another limitation was the use of a single experimental model for CPM and TSP. Different paradigms for CPM have been conducted in the pediatric population.^17^ Temporal summation can also be assessed by applying a series of heat-pain stimuli of the same temperature (eg, 47°C).^50^ It is unknown whether the use of another experimental pain procedure would have produced different results. Although the main strength of the current experimental procedure allows to elicit and measure multiple pain modulation responses, adding another CPM paradigm and TSP paradigm may further strengthen our findings.

In conclusion, this study highlights the heterogeneity in facilitatory and inhibitory pain modulation responses in a large sample of pediatric patients with chronic pain. Furthermore, chronic pediatric pain was found to be associated with cold hyperalgesia, and a subgroup of patients was identified to display increased TSP and reduced inhibitory CPM efficacy. Future studies with a longitudinal design are required to replicate the clusters identified and to determine is these clusters predict the development of diffuse widespread pain. Moreover, such studies will need to pay attention to the methodological characteristics of the experimental paradigms conducted.

## Disclosures

The authors have no conflicts of interest to declare.

## Appendix A. Supplemental digital content

Supplemental digital content associated with this article can be found online at http://links.lww.com/PR9/A172.

## Supplementary Material

SUPPLEMENTARY MATERIAL

## References

[1] Abad F Diaz-Gomez NM Rodriguez I Perez R Delgado JA. Subclinical pain and thermal sensory dysfunction in children and adolescents with Type 1 diabetes mellitus. Diabet Med 2002;19:827–31. 12358869 10.1046/j.1464-5491.2002.00793.x

[2] Bettini EA Moore K Wang Y Hinds PS Finkel JC. Association between pain sensitivity, central sensitization, and functional disability in adolescents with joint hypermobility. J Pediatr Nurs 2018;42:34–8. 30219297 10.1016/j.pedn.2018.06.007

[3] Birnie KA Caes L Wilson AC Williams SE Chambers CT. A practical guide and perspectives on the use of experimental pain modalities with children and adolescents. Pain Manag 2014;4:97–111. 24641434 10.2217/pmt.13.72 PMC4110966

[4] Birnie KA Petter M Boerner KE Noel M Chambers CT. Contemporary use of the cold pressor task in pediatric pain research: a systematic review of methods. J Pain 2012;13:817–26. 22846592 10.1016/j.jpain.2012.06.005

[5] Blankenburg M Boekens H Hechler T Maier C Krumova E Scherens A Magerl W Aksu F Zernikow B. Reference values for quantitative sensory testing in children and adolescents: developmental and gender differences of somatosensory perception. PAIN 2010;149:76–88. 20138430 10.1016/j.pain.2010.01.011

[6] Brandow AM Stucky CL Hillery CA Hoffmann RG Panepinto JA. Patients with sickle cell disease have increased sensitivity to cold and heat. Am J Hematol 2013;88:37–43. 23115062 10.1002/ajh.23341 PMC3552380

[7] Chretien R Lavoie S Chalaye P de Vette E Counil FP Dallaire F Lafrenaye S. Reduced endogenous pain inhibition in adolescent girls with chronic pain. Scand J Pain 2018;18:711–17. 30007060 10.1515/sjpain-2018-0071

[8] de Tommaso M Sciruicchio V Delussi M Vecchio E Goffredo M Simeone M Barbaro MGF. Symptoms of central sensitization and comorbidity for juvenile fibromyalgia in childhood migraine: an observational study in a tertiary headache center. J Headache Pain 2017;18:59. 28560539 10.1186/s10194-017-0764-8 PMC5449358

[9] Evans S Payne LA Seidman L Lung K Zeltzer L Tsao JCI. Maternal anxiety and children's laboratory pain: the mediating role of solicitousness. Children 2016;3:10. 10.3390/children3020010 PMC4934565 27417248

[10] Evans S Seidman LC Lung KC Zeltzer LK Tsao JC. Sex differences in the relationship between maternal fear of pain and children's conditioned pain modulation. J Pain Res 2013;6:231–8. 23569396 10.2147/JPR.S43172 PMC3615838

[11] Farrar JT Young JP Jr LaMoreaux L Werth JL Poole RM. Clinical importance of changes in chronic pain intensity measured on an 11-point numerical pain rating scale. PAIN 2001;94:149–58. 11690728 10.1016/S0304-3959(01)00349-9

[12] Ferland CE Teles AR Ingelmo P Saran N Marchand S Ouellet JA. Blood monoamines as potential biomarkers for conditioned pain modulation efficacy: an exploratory study in paediatrics. Eur J Pain 2019;23:327–40. 30125426 10.1002/ejp.1307

[13] Giehl J Meyer-Brandis G Kunz M Lautenbacher S. Responses to tonic heat pain in the ongoing EEG under conditions of controlled attention. Somatosensory Mot Res 2014;31:40–8. 10.3109/08990220.2013.837045 24320554

[14] Goffaux P Lafrenaye S Morin M Patural H Demers G Marchand S. Preterm births: can neonatal pain alter the development of endogenous gating systems? Eur J Pain 2008;12:945–51. 18308597 10.1016/j.ejpain.2008.01.003

[15] Hair JF. Multivariate data analysis: A global perspective. Upper Saddle River, NJ; London: Pearson Education, 2010.

[16] Holley AL Wilson AC Palermo TM. Predictors of the transition from acute to persistent musculoskeletal pain in children and adolescents: a prospective study. PAIN 2017;158:794–801. 28151835 10.1097/j.pain.0000000000000817 PMC5393939

[17] Hwang PS Ma ML Spiegelberg N Ferland CE. Current methodological approaches in conditioned pain modulation assessment in pediatrics. J Pain Res 2017;10:2797–802. 29263694 10.2147/JPR.S150857 PMC5732558

[18] King S Chambers CT Huguet A MacNevin RC McGrath PJ Parker L MacDonald AJ. The epidemiology of chronic pain in children and adolescents revisited: a systematic review. PAIN 2011;152:2729–38. 22078064 10.1016/j.pain.2011.07.016

[19] Le S Josse J Husson F. FactoMineR: an R package for multivariate analysis. J Stat Softw 2008;25:1–18.

[20] Lewandowski Holley A Wilson AC Cho E Palermo TM. Clinical phenotyping of youth with new-onset musculoskeletal pain: a controlled cohort study. Clin J Pain 2017;33:28–36. 27340913 10.1097/AJP.0000000000000371 PMC5140689

[21] Mansour AR Farmer MA Baliki MN Apkarian AV. Chronic pain: the role of learning and brain plasticity. Restor Neurol Neurosci 2014;32:129–39. 23603439 10.3233/RNN-139003 PMC4922795

[22] Morris MC Walker LS Bruehl S Stone AL Mielock AS Rao U. Impaired conditioned pain modulation in youth with functional abdominal pain. PAIN 2016;157:2375–81. 27389918 10.1097/j.pain.0000000000000660 PMC5028273

[23] Nahman-Averbuch H Leon E Hunter BM Ding L Hershey AD Powers SW King CD Coghill RC. Increased pain sensitivity but normal pain modulation in adolescents with migraine. PAIN 2019;160:1019–28. 30624343 10.1097/j.pain.0000000000001477

[24] Nahman-Averbuch H Nir RR Sprecher E Yarnitsky D. Psychological factors and conditioned pain modulation: a meta-analysis. Clin J Pain 2016;32:541–54. 26340657 10.1097/AJP.0000000000000296

[25] Nir RR Sinai A Moont R Harari E Yarnitsky D. Tonic pain and continuous EEG: prediction of subjective pain perception by alpha-1 power during stimulation and at rest. Clin Neurophysiol 2012;123:605–12. 21889398 10.1016/j.clinph.2011.08.006

[26] Nir RR Yarnitsky D. Conditioned pain modulation. Curr Opin Support Palliat Care 2015;9:131–7. 25699686 10.1097/SPC.0000000000000126

[27] Ocay DD Larche CL Betinjane N Jolicoeur A Beaulieu MJ Saran N Ouellet JA Ingelmo PM Ferland CE. Phenotyping chronic musculoskeletal pain in male and female adolescents: psychosocial profiles, somatosensory profiles and pain modulatory profiles. J Pain Res 2022;15:591–612. 35250304 10.2147/JPR.S352607 PMC8892739

[28] Ocay DD Loewen A Premachandran S Ingelmo PM Saran N Ouellet JA Ferland CE. Psychosocial and psychophysical assessment in paediatric patients and young adults with chronic back pain: a cluster analysis. Eur J Pain 2022;26:855–72. 35090183 10.1002/ejp.1912 PMC9304192

[29] Pas R Rheel E Van Oosterwijck S Leysen L Van De Vijver E Nijs J Ickmans K Meeus M. Endogenous pain modulation in children with functional abdominal pain disorders. PAIN 2019;160:1883–90. 31335656 10.1097/j.pain.0000000000001566

[30] Petter M Chambers CT MacLaren Chorney J. The effects of mindfulness-based attention on cold pressor pain in children. Pain Res Manag 2013;18:39–45. 23457685 10.1155/2013/857045 PMC3665436

[31] Popescu A LeResche L Truelove EL Drangsholt MT. Gender differences in pain modulation by diffuse noxious inhibitory controls: a systematic review. PAIN 2010;150:309–18. 20557999 10.1016/j.pain.2010.05.013

[32] Potvin S Marchand S. Pain facilitation and pain inhibition during conditioned pain modulation in fibromyalgia and in healthy controls. PAIN 2016;157:1704–10. 27045524 10.1097/j.pain.0000000000000573

[33] Potvin S Paul-Savoie E Morin M Bourgault P Marchand S. Temporal summation of pain is not amplified in a large proportion of fibromyalgia patients. Pain Res Treat 2012;2012:938595. 22701791 10.1155/2012/938595 PMC3372092

[34] Potvin S Stip E Tempier A Pampoulova T Bentaleb LA Lalonde P Lipp O Goffaux P Marchand S. Pain perception in schizophrenia: no changes in diffuse noxious inhibitory controls (DNIC) but a lack of pain sensitization. J Psychiatr Res 2008;42:1010–16. 18093615 10.1016/j.jpsychires.2007.11.001

[35] Scharff L Langan N Rotter N Scott-Sutherland J Schenck C Tayor N McDonald-Nolan L Masek B. Psychological, behavioral, and family characteristics of pediatric patients with chronic pain: a 1-year retrospective study and cluster analysis. Clin J Pain 2005;21:432–8. 16093749 10.1097/01.ajp.0000130160.40974.f5

[36] Schulz E May ES Postorino M Tiemann L Nickel MM Witkovsky V Schmidt P Gross J Ploner M. Prefrontal gamma oscillations encode tonic pain in humans. Cereb Cortex 2015;25:4407–14. 25754338 10.1093/cercor/bhv043 PMC4816790

[37] Schurman JV Danda CE Friesen CA Hyman PE Simon SD Cocjin JT. Variations in psychological profile among children with recurrent abdominal pain. J Clin Psychol Med Settings 2008;15:241–51. 19104969 10.1007/s10880-008-9120-0

[38] Sherman AL Morris MC Bruehl S Westbrook TD Walker LS. Heightened temporal summation of pain in patients with functional gastrointestinal disorders and history of trauma. Ann Behav Med 2015;49:785–92. 25967582 10.1007/s12160-015-9712-5 PMC4636446

[39] Soee AB Thomsen LL Kreiner S Tornoe B Skov L. Altered pain perception in children with chronic tension-type headache: is this a sign of central sensitisation? Cephalalgia 2013;33:454–62. 23439572 10.1177/0333102413476371

[40] Teles AR Ocay DD Bin Shebreen A Tice A Saran N Ouellet JA Ferland CE. Evidence of impaired pain modulation in adolescents with idiopathic scoliosis and chronic back pain. Spine J 2019;19:677–86. 30343045 10.1016/j.spinee.2018.10.009

[41] Thastum M Zachariae R Herlin T. Pain experience and pain coping strategies in children with juvenile idiopathic arthritis. J Rheumatol 2001;28:1091–8. 11361195

[42] Tousignant-Laflamme Y Page S Goffaux P Marchand S. An experimental model to measure excitatory and inhibitory pain mechanisms in humans. Brain Res 2008;1230:73–9. 18652808 10.1016/j.brainres.2008.06.120

[43] Tsao JC Evans S Seidman LC Zeltzer LK. Experimental pain responses in children with chronic pain and in healthy children: how do they differ? Pain Res Manag 2012;17:103–9. 22518373 10.1155/2012/592108 PMC3393051

[44] Tsao JC Seidman LC Evans S Lung KC Zeltzer LK Naliboff BD. Conditioned pain modulation in children and adolescents: effects of sex and age. J Pain 2013;14:558–67. 23541066 10.1016/j.jpain.2013.01.010 PMC3672325

[45] Tsze DS Hirschfeld G von Baeyer CL Suarez LE Dayan PS. Changes in pain score associated with clinically meaningful outcomes in children with acute pain. Acad Emerg Med 2019;26:1002–13. 30636350 10.1111/acem.13683 PMC6626586

[46] Vaegter HB Graven-Nielsen T. Pain modulatory phenotypes differentiate subgroups with different clinical and experimental pain sensitivity. PAIN 2016;157:1480–8. 26963852 10.1097/j.pain.0000000000000543

[47] van den Bosch GE van Dijk M Tibboel D Valkenburg AJ. Thermal quantitative sensory testing in healthy Dutch children and adolescents standardized test paradigm and Dutch reference values. BMC Pediatr 2017;17:77. 28302148 10.1186/s12887-017-0827-7 PMC5356312

[48] Vervoort T Trost Z Van Ryckeghem DML. Children's selective attention to pain and avoidance behaviour: the role of child and parental catastrophizing about pain. PAIN 2013;154:1979–88. 23792243 10.1016/j.pain.2013.05.052

[49] Wager J Zernikow B Darlington A Vocks S Hechler T. Identifying subgroups of paediatric chronic pain patients: a cluster-analytic approach. Eur J Pain 2014;18:1352–62. 24700548 10.1002/j.1532-2149.2014.497.x

[50] Walker LS Sherman AL Bruehl S Garber J Smith CA. Functional abdominal pain patient subtypes in childhood predict functional gastrointestinal disorders with chronic pain and psychiatric comorbidities in adolescence and adulthood. PAIN 2012;153:1798–806. 22721910 10.1016/j.pain.2012.03.026 PMC3413740

[51] Weyandt LL Clarkin CM Holding EZ May SE Marraccini ME Gudmundsdottir BG Shepard E Thompson L. Neuroplasticity in children and adolescents in response to treatment intervention: a systematic review of the literature. Clin Translational Neurosci 2020;4:2514183X20974231.

[52] Williams AE Heitkemper M Self MM Czyzewski DI Shulman RJ. Endogenous inhibition of somatic pain is impaired in girls with irritable bowel syndrome compared with healthy girls. J Pain 2013;14:921–30. 23685184 10.1016/j.jpain.2013.03.003 PMC3759538

[53] Yarnitsky D. Role of endogenous pain modulation in chronic pain mechanisms and treatment. PAIN 2015;156 suppl 1:S24–31. 25789433 10.1097/01.j.pain.0000460343.46847.58

[54] Yarnitsky D Bouhassira D Drewes AM Fillingim RB Granot M Hansson P Landau R Marchand S Matre D Nilsen KB Stubhaug A Treede RD Wilder-Smith OH. Recommendations on practice of conditioned pain modulation (CPM) testing. Eur J Pain 2015;19:805–6. 25330039 10.1002/ejp.605

[55] Zohsel K Hohmeister J Flor H Hermann C. Somatic pain sensitivity in children with recurrent abdominal pain. Am J Gastroenterol 2008;103:1517–23. 18510619 10.1111/j.1572-0241.2008.01911.x

[56] Zohsel K Hohmeister J Oelkers-Ax R Flor H Hermann C. Quantitative sensory testing in children with migraine: preliminary evidence for enhanced sensitivity to painful stimuli especially in girls. PAIN 2006;123:10–18. 16495010 10.1016/j.pain.2005.12.015

