# Supplementary material for: Clusters of facilitatory and inhibitory conditioned pain modulation responses in a large sample of children, adolescents, and young adults with chronic pain
Source: Pain Rep. 2022 Oct 4;7(6):e1032. doi: 10.1097/PR9.0000000000001032 (PMC9534368; doi:10.1097/PR9.0000000000001032)
Supplement: SUPPLEMENTARY MATERIAL [file painreports-7-e1032-s001.pdf]

**Supplementary Table 1. Description of sample population by study**

| **Study** | |
|---|---|
| A08-M71-14B (n=55) | Adolescents with idiopathic scoliosis scheduled to undergo spinal fusion surgery (10-21 years old). The data used were of the baseline timepoint |
| A11-M62-15B (n=181) | Cross-sectional study investigating chronic back pain (10-21 years old).<br>Arthritic=5<br>Disc protrusion=7<br>Mechanical back pain=14<br>Scoliosis=103<br>Spondylolysis=13<br>Tight hamstrings=7<br>Non-specific=32 |
| A09-M17-17B (n=221) | Cross-sectional study investigating chronic primary or secondary pain (10-18 years old).<br>Amplified musculoskeletal pain syndrome=1<br>Arthritic back pain=2<br>Asperger's syndrome=1<br>Chronic Postsurgical and Posttraumatic Pain=5<br>Chronic widespread pain=20<br>Coccydynia=1<br>Complex regional pain syndrome=3<br>Disc protrusion=6<br>Ehler Danlos Syndrome=3<br>Juvenile idiopathic arthritis=2<br>Kyphosis=2<br>Mechanical back pain=2<br>Merosin-deficient congenital muscular dystrophy=1<br>Osteogenesis imperfecta=8<br>Osteoporosis=1<br>Patellofemoral pain syndrome=4<br>Pectus excavatum=1<br>Planovalgus=1<br>Radiculopathy=2<br>Scoliosis=71<br>Spondylolysis=10<br>Syringomyelia=1<br>Tight hamstrings=1<br>N/A=72 |
| 2019-4887 (n=151) | Patients prospectively followed at a center for complex pain (8-18 years old). The data used were of the baseline timepoint, prior to clinical care.<br>Chronic cancer-related pain=1<br>Chronic postsurgical and posttraumatic pain=15<br>Chronic primary headache or orofacial pain=19<br>Chronic primary musculoskeletal pain=35<br>Chronic primary visceral pain=10<br>Chronic secondary headache or orofacial pain=7<br>Chronic secondary musculoskeletal pain=19<br>Chronic secondary neuropathic pain=6<br>Chronic secondary visceral pain=5<br>Chronic widespread pain=25<br>Complex regional pain syndrome=9 |

**Supplementary Table 2. Within cohort differences and correlations**

| Variable | HPT | T50 | TSP1 | CS | TSP2 | CPM |
|---|---|---|---|---|---|---|
| ***Age** | 0.038 (0.329) | 0.137 (<0.001) | -0.010 (0.791) | -0.086 (0.027) | -0.028 (0.476) | 0.038 (0.333) |
| **[†]Gender** | 4.571 (0.033) | 1.089 (0.297) | 0.178 (0.673) | 0.254 (0.615) | 0.087 (0.768) | 1.941 (0.164) |
| ***Pain prior to CPM assessment** | -0.078 (0.045) | -0.004 (0.911) | -0.022 (0.572) | 0.036 (0.355) | -0.071 (0.068) | 0.037 (0.336) |
| ***Test temperature** | | | 0.172 (<0.001) | | 0.157 (<0.001) | -0.031 (0.421) |

Data is presented as rho-value (p-value), except for gender which is presented as F-value (p-value)
*Spearman correlation. †One-way ANOVA test.
